# Genetic, Sociodemographic and Clinical Determinants of COVID-19 Severity in the Republic of Srpska: Exploring Potential Links with Neanderthal-Derived Variants

**DOI:** 10.3390/biomedicines14020478

**Published:** 2026-02-22

**Authors:** Milena Dubravac Tanasković, Biljana Mijović, Jovan Kulić, Bojan Joksimović, Kristina Drašković-Mališ, Srđan Mašić, Jelena Vladičić-Mašić, Ljiljana Krsmanović, Danijela Radulović, Nikolina Elez-Burnjaković

**Affiliations:** 1 Faculty of Medicine Foča, University of East Sarajevo, 73300 Foča, Bosnia and Herzegovina; menadub@gmail.com (M.D.T.); biljana.mijovic@ues.rs.ba (B.M.); kulicjovan@yahoo.com (J.K.); bojannjoksimovic@gmail.com (B.J.); kristina.dr995@gmail.com (K.D.-M.); srdjan.masic@ues.rs.ba (S.M.); jelenavladicmasic@gmail.com (J.V.-M.); ljiljanakrsmanovic85@gmail.com (L.K.); danijelarmf@yahoo.com (D.R.); 2 University Hospital Foča, 73300 Foča, Bosnia and Herzegovina

**Keywords:** COVID-19, SARS-CoV-2, Neanderthal-derived variants, *OAS3*, *LZTFL1*, disease severity, Republic of Srpska

## Abstract

**Background/Objectives:** COVID-19 severity is influenced by a complex interplay between host, viral, and environmental factors. Emerging evidence suggests that Neanderthal-derived genetic variants may influence the progression and severity of SARS-CoV-2 infection. This study aimed to evaluate the association between selected Neanderthal-derived variants and COVID-19 severity in the population of the Republic of Srpska, considering relevant clinical, sociodemographic, and lifestyle factors. **Methods:** This multicentric cross-sectional study included 402 participants, classified as healthy or SARS-CoV-2-positive individuals. A total of 378 COVID-19-positive participants were further stratified according to disease severity and hospitalization status. All individuals were genotyped for the Neanderthal-derived *OAS3* rs1156361 (C/T) and *LZTFL1* rs35044562 (A/G) variants. Detailed sociodemographic, clinical, and lifestyle data were also collected. **Results:** A higher frequency of the *LZTFL1* rs35044562 AG genotype was observed among hospitalized patients compared with non-hospitalized individuals (36.8% vs. 20.9%; *p* = 0.005), while the AA genotype was more prevalent among non-hospitalized patients (77.3% vs. 63.2%, *p* = 0.015). Multivariable logistic analysis showed that carriers of the *LZTFL1* AG genotype had a higher chance of hospitalization compared to AA carriers (adjusted OR = 1.372, 95% CI = 0.763–6.383, and *p* = 0.021). Hospitalized patients more frequently carried the combined CT (*OAS3*) and AG (*LZTFL1*) genotypes, supporting a potential synergistic effect. Several sociodemographic factors, including age, sex, education, employment, and urban residence, were also associated with COVID-19 severity, while no significant associations were observed in allele-based analyses. **Conclusions:** *LZTFL1* gene polymorphisms may influence COVID-19 severity, with heterozygote-specific and combined risk effects observed. These preliminary findings are exploratory and require validation in larger cohorts, but may guide future studies and targeted interventions in high-risk groups.

## 1. Introduction

As the boundaries between urban and natural spaces more frequently overlap and interspecies interactions intensify, zoonoses are becoming a growing public health concern [1]. Numerous diseases that are a consequence of the spillover phenomenon have historically shaped human demographic and social structures [1,2]. The most recent COVID-19 pandemic caused by severe acute respiratory syndrome virus 2 (SARS-CoV-2) has once again demonstrated the devastating potential of zoonotic agents in the modern world [3]. To date, according to the World Health Organization (WHO) database, nearly 780 million confirmed cases of COVID-19 have been registered, and the number of deaths potentially associated with this disease has reached approximately 7 million [4]. COVID-19 presents with a wide range of symptoms, from mild to life-threatening [5]. Its severity is a result of the complex interaction of viral, host, and environmental factors [6,7]. From the very beginning of the COVID-19 pandemic, it became evident that advanced age, male sex, lifestyle and certain comorbidities represent significant predictors of disease severity [6,7,8,9,10]. However, these risk factors cannot fully explain the considerable individual variability observed in the clinical course and potential outcomes of the infection. For instance, severe forms of the disease have been reported even among younger individuals without underlying comorbidities [6,7,11]. Additionally, several family clusters of severe cases have been identified, along with regional variations in the occurrence of certain clinical manifestations of COVID-19 [6,7,11]. This suggests that genetic variability, particularly in individual susceptibility and immune response, plays a key role in determining disease severity [8,9,10].

In recent years, multiple studies have provided important findings suggesting that archaic genetic heritage, particularly from Neanderthals, can influence immune functions, especially when it comes to antiviral immune response [12,13]. Notably, several studies have identified associations between Neanderthal-derived genetic variants and COVID-19 [14,15,16,17,18,19].

While some Neanderthal-derived variants have been associated with increased disease severity, others appear to confer protection. For instance, a protective haplotype on chromosome 12, also of Neanderthal origin, encompassing the *OAS1*, *OAS2*, and *OAS3* genes, has been associated with reduced risk of severe COVID-19 through enhanced antiviral activity [14,15,20]. This haplotype, spanning approximately 75 kilobases, is relatively common across global populations outside of Africa [17]. The 2′–5′-oligoadenylate synthetase (OAS) family comprises interferon-induced, double-stranded RNA-dependent enzymes that play a critical role in immune-mediated host defense against viral infections, by contributing to the degradation of viral RNA and the inhibition of viral replication [14,15]. While all OAS family members share similar functions, OAS3 plays a central role by recognizing longer viral RNA molecules and initiating their degradation [21,22]. Acting as the primary viral RNA sensor, OAS3 serves as the “detector,” whereas OAS1 and OAS2 function as downstream “effectors” [21,22]. During the COVID-19 pandemic, *OAS* gene cluster polymorphisms were highlighted for their protective role against severe SARS-CoV-2 infection. The *OAS1/2/3* haplotype is estimated to reduce the risk of severe disease by ~23%, with functionally important alleles including *OAS1* rs10774671 and *OAS3* rs1156361 [15,16,20].

One of the most remarkable findings was reported by Zeberg and Pääbo, who identified several genomic regions within the 3p21.31 locus that are associated with a 60% increased likelihood of hospitalization due to COVID-19 [18]. This so-called risk haplotype includes a cluster of genes (*SLC6A20*, *LZTFL1*, *CCR9*, *FYCO1*, *CXCR6*, and *XCR1*) involved in immune regulation and inflammation [14,18]. It is present in approximately 30% of South Asians and 63% of individuals from Bangladesh, but occurs less frequently in Europeans (8%) and Latin Americans (4%), and it is rare or absent in East Asian and African populations [14,17]. The immune response mediated by genes located on this haplotype could be too aggressive, leading to a potentially fatal immune reaction such is seen in people who have developed severe forms of COVID-19 [14,18]. Several variants within this region (rs35044562, rs73064425, and rs67959919), particularly in the *LZTFL1* gene, have been identified as key contributors to severe disease phenotypes [17,18]. LZTFL1 acts as a tumor suppressor by regulating epithelial-to-mesenchymal transition (EMT) through Wnt/β-catenin and TGF-β signaling. EMT can be induced by SARS-CoV-2 in respiratory epithelial cells and may underline the risk associated with the 3p21.31 locus [20,21].

Beyond evaluating the impact of previously identified epidemiological, clinical, and sociodemographic factors associated with the development and severity of SARS-CoV-2 infection, this study aimed to assess the frequency of selected genetic variants in the *LZTFL1* (rs35044562) and *OAS3* (rs1156361) genes within the population of the Republic of Srpska. Furthermore, we investigated their potential associations with COVID-19 severity, including the risk of hospitalization among SARS-CoV-2-infected individuals.

## 2. Materials and Methods

### 2.1. Study Design and Population

This exploratory multicentric cross-sectional study was conducted from December 2021 to January 2022 across multiple cities and municipalities of the Republic of Srpska, Bosnia and Herzegovina, in parallel with a SARS-CoV-2 seroprevalence survey. A stratified random sample was drawn from individuals registered with family medicine practices, ensuring proportional representation of urban (30%) and non-urban (70%) areas. A linear sampling method with a random start point and fixed interval was used, providing equal selection probability without replacement.

Data were collected using patients’ medical records obtained from family medicine practitioners, the WHO seroprevalence questionnaire, and a specifically developed population-genetic questionnaire. The study analyzed participants’ sociodemographic characteristics (sex, age, education level, occupation, and place of residence), clinical information related to SARS-CoV-2 infection (symptoms, hospitalization, and testing), vaccination history, comorbidities, ongoing therapies, and lifestyle-related factors. All recruited participants were previously tested for SARS-CoV-2 by RT-PCR using a nasopharyngeal swab and had antibody titer data available from the seroprevalence study.

A total of 402 enrolled participants were evaluated according to the presence and severity of clinical manifestations and categorized as 24 healthy individuals and 378 COVID-19-positive patients. The primary analytical focus of the study was on SARS-CoV-2-positive individuals, who were further divided into 77 asymptomatic and 301 symptomatic cases. Among the symptomatic participants, 76 required hospitalization due to the severity of their symptoms, while 225 were treated as outpatients. Given the limited number of COVID-19-negative individuals in the population at the time of sampling, they were included primarily as a reference group for exploratory susceptibility analyses.

### 2.2. Sample Collection, DNA Extraction and Genotyping

Laboratory analyses were carried out between May and December 2023. Peripheral blood samples were collected in EDTA-containing tubes and immediately stored at −86 °C until further processing. Genomic DNA was extracted from whole blood using commercially available DNA extraction kits (DNaeasy Blood and Tissue Kit, Qiagen, Germany and PureLink™ Genomic DNA Mini Kit, Invitrogen, Waltham, MA, USA), and with the salting-out method to ensure high purity and yield. The concentration and quality of the isolated DNA were assessed using a commercial kit (Qubit dsDNA BR Assay Kit, Thermo Fisher Scientific, Waltham, MA, USA) and a Qubit 4 fluorometer (Thermo Fisher Scientific, Waltham, MA, USA). Genotyping was performed using the TaqMan™ SNP Genotyping Assay (Applied Biosystems, Thermo Fisher Scientific, Waltham, MA, USA), following the manufacturer’s protocol. The analysis was carried out on a StepOnePlus Real-Time PCR System (Applied Biosystems, Thermo Fisher Scientific, Waltham, MA, USA). The study analyzed the following single-nucleotide polymorphisms (SNPs): *OAS3* (rs1156361; C/T) and *LZTFL1* (rs35044562; A/G).

### 2.3. Statistical Analysis

Descriptive and analytical statistical methods were applied in the study. Continuous variables were summarized as measures of central tendency and variability (arithmetic mean ± standard deviation, median, as well as minimum and maximum values), while categorical variables were expressed as relative frequencies. Analytical methods were used to determine statistically significant differences between groups. Numerical variables were analyzed using Student’s *t*-test for paired samples or one-way ANOVA, depending on the number of groups. Categorical variables were assessed with nonparametric tests such as the chi-square test, Fisher’s exact test, and Kruskal–Wallis, as appropriate. Risk factor analysis was performed using binary logistic regression. The *p*-value of <0.050 was considered statistically significant. All analyses were performed using SPSS software, version 23.0 (Statistical Package for Social Sciences SPSS 23.0 Inc., Chicago, IL, USA).

### 2.4. Ethical Statement

The study was approved by the Ethics Committee of the Faculty of Medicine Foča, University of East Sarajevo (Decision number: 01-2-8 dated 6 November 2020). All participants provided written informed consent after a detailed explanation of the study protocol. Participants who refused to participate were not included in the study, nor were the members of their families. The research was conducted in accordance with the Helsinki Declaration and the principles of Good Clinical Practice.

## 3. Results

### 3.1. Sociodemographic, Epidemiological, and Clinical Characteristics

Baseline sociodemographic, epidemiological, and clinical characteristics of 402 study participants are presented in Supplementary Table S1.

Comparison of 77 (20.4%) asymptomatic and 301 (79.6%) symptomatic COVID-19 positive individuals showed a significant difference in age distribution between groups, driven by a higher proportion of individuals aged 8–34 years in the asymptomatic group (31.2% vs. 17.6%; *p* = 0.019). Symptomatic patients had higher education levels (33.2% vs. 13.0%; *p* < 0.001), were more frequently regularly employed (55.5% vs. 32.5%; *p* < 0.001), and were more often urban residents (66.1% vs. 33.8%; *p* < 0.001). SARS-CoV-2 IgG seropositivity was lower among asymptomatic individuals (75.5% vs. 91.1%; *p* = 0.015). Occasional sports activity was less common in this group (15.6% vs. 27.6%; *p* = 0.019). Regarding comorbidities, obesity was less prevalent among asymptomatic patients (15.6% vs. 29.6%; *p* = 0.013), while hypercholesterolemia showed borderline significance (*p* = 0.050). The remaining variables showed no significant differences across groups (Table 1).

The aforementioned characteristics were also compared between non-hospitalized (n = 225, 74.8%) and hospitalized (n = 76, 25.2%) symptomatic COVID-19 patients. Hospitalized individuals were significantly older (57.33 ± 12.03 vs. 45.27 ± 15.04 years; *p* < 0.001), with a higher proportion in the 50–83 year age group (71.1% vs. 38.2%; *p* < 0.001). A higher proportion of hospitalized patients were male (52.6% vs. 36.9%; *p* = 0.016), and they had higher BMI values (28.03 ± 2.79 vs. 25.86 ± 3.24 kg/m^2^; *p* = 0.005). Regular employment was less common among hospitalized patients (40.8% vs. 60.4%; *p* = 0.003), while smoking was reported less frequently in this group (10.5% vs. 28.0%; *p* = 0.002). Several comorbidities were more prevalent among hospitalized individuals, including diabetes mellitus (19.7% vs. 8.0%; *p* = 0.005), hypertension (57.9% vs. 24.9%; *p* < 0.001), hypercholesterolemia (32.9% vs. 17.8%; *p* = 0.006), obesity (43.4% vs. 24.9%; *p* = 0.002), and cardiovascular diseases (26.3% vs. 9.8%; *p* < 0.001). No notable group differences were identified for the remaining variables (Table 2).

### 3.2. Genotype and Allele Frequencies and Their Association with COVID-19 Susceptibility and Severity

Neanderthal variants revealed a distribution in the Hardy–Weinberg equilibrium (HWE) in COVID-19 positive patients. Indeed, the X^2^_HWE_ *p* values for the rs1156361 and rs35044562 variants did not reach statistical significance in the patient group; these two variants were confirmed to be in HWE (X^2^ _HWE_ = 0.0549, *p* = 0.8148; X^2^ _HWE_ = 2.2866, *p* = 0.1305) (Supplementary Table S2). The genotype frequencies correspond to those expected from the allele frequencies. The obtained allele frequencies were compared with the allele frequencies for the European population. The comparison of our results with data from the project “ALFA: Allele Frequency Aggregator” shows that the frequency of alleles corresponds to the average frequency in the European population. No statistically significant differences were observed (Supplementary Table S3). There is no evidence of selection, mutation, migration, inbreeding, or genotyping errors that would significantly disrupt the equilibrium, and the data appear reliable. Therefore, the loci are not under evolutionary pressure.

Table 3 examines *OAS3* rs1156361 and *LZTFL1* rs35044562 genotypes and alleles frequences and their association with symptom manifestation in COVID-19-positive individuals. No significant differences were noticed for *OAS3* and *LZTFL1* genotypes or allele frequencies, with none of the genotypes identified as independent risk factors of the occurrence of symptoms.

The potential impact of *OAS3* rs1156361 and *LZTFL1* rs35044562 variants on hospitalization risk in symptomatic COVID-19 patients was assessed through genotype and allele frequency analysis (Table 4). The frequencies of *OAS3* rs1156361 genotypes and alleles showed no significant differences, with multivariate analysis confirming a lack of association with hospitalization risk. For *LZTFL1* rs35044562, the AA genotype was more prevalent among nonhospitalized (77.3% vs. 63.2%, *p* = 0.015), while the AG genotype was more frequent in hospitalized patients (36.8% vs. 20.9%; *p* = 0.005). Multivariate analysis showed that the carriers of the AG genotype had a 1.3 times higher chance of being hospitalized when compared to the referent AA genotype (AOR = 1.372, 95% CI = 0.763–6.383, *p* = 0.021). The GG genotype frequency did not differ between the groups. Although differences in allele frequencies between groups were observed, they did not reach statistical significance (*p* = 0.058).

### 3.3. Association of Different Genetic Inheritance Models with COVID-19 Severity

Comparing genotype distribution under the dominant (AA vs. AG + GG) and overdominant model (AG vs. AA + GG) revealed significant differences between non-hospitalized and hospitalized COVID-19 patients (*p* = 0.015 and *p* = 0.010 respectively), indicating that the AG genotype may be associated with higher risk of hospitalization, while AA genotype was more frequent among non-hospitalized patients indicating its possible protective role against severe disease. Additional multivariate analysis showed that the AG + GG genotype was significantly associated with hospitalization (AOR = 1.101, 95% CI = 0.690–4.229; *p* = 0.022) (Table 5).

### 3.4. Combined Genotype Effects

The association of combined *OAS3* rs1156361 and *LZTFL1* rs35044562 genotypes was evaluated across different clinical outcomes. While no significant associations were observed between asymptomatic and symptomatic COVID-19 positive participants, the CT/AG genotype combination was significantly associated with COVID-19-related hospitalization (6.2% among non-hospitalized vs. 18.4% among hospitalized patients; *p* = 0.020). All other genotype combinations showed no statistically significant differences between groups (Supplementary Table S4).

### 3.5. Exploratory Analysis of Genetic Susceptibility to SARS-CoV-2 Infection

The genotype and allele frequencies of *OAS3* rs1156361 and *LZTFL1* rs35044562 polymorphisms in healthy individuals and COVID-19 positive patients, as well as their associations with susceptibility to SARS-CoV-2 infection under different allelic models, are summarized in Table 6 and Table 7.

While certain genotype distributions differed between the groups, the small number of healthy individuals limits statistical power and precludes confirming conclusions regarding genetic susceptibility. These analyses therefore serve as an exploratory context, whereas the primary findings of the study relate to disease severity and hospitalization outcomes among infected individuals.

## 4. Discussion

The severity of the clinical course of SARS-CoV-2 infection is influenced by a complex interplay of clinical, genetic, demographic and social factors. Within this multifactorial framework, our analysis aimed to identify additional determinants associated with disease progression. In our cohort, several sociodemographic, epidemiological and clinical characteristics emerged as important risk factors for severe COVID-19.

Sex and age were significant determinants of COVID-19 outcomes in our cohort. Male sex was associated with more severe disease and higher hospitalization rates, in line with previous reports indicating worse clinical outcomes in men, including increased rates of hospitalization, intensive care unit admission, and mortality [23,24,25,26,27]. Younger adults were more often asymptomatic, whereas the risk of severe disease and hospitalization increases markedly with age, with individuals aged ≥50 years accounting for the majority of hospitalized cases, consistent with previous population-based studies and meta-analyses highlighting age as a risk factor for severe disease [28,29,30,31,32,33,34,35].

Sociodemographic factors, including education, employment, and urban residence, were associated with COVID-19 outcomes in our cohort, with some findings differing from emerging evidence from the literature. Lower educational attainment was more common among asymptomatic individuals, likely reflecting differences in health literacy, symptom recognition, or reporting behavior rather than a direct biological effect [36,37]. Employment status showed complex associations. Hospitalized patients were more often unemployed, which is consistent with associations between unemployment, reduced healthcare access, and higher comorbidity burden, while asymptomatic infections were also more frequent among those not employed, suggesting that work-related exposure may modulate infection intensity or symptom manifestation [36,37,38]. Urban residence was generally associated with infection, yet asymptomatic cases were less likely to reside in urban areas, implying that higher population density may contribute to symptomatic disease through increased exposure [39,40].

In terms of lifestyle, smoking was more common among non-hospitalized individuals, illustrating the “smoking paradox”. This paradox refers to the apparently contradictory observation that smokers may exhibit better short-term clinical outcomes in certain acute conditions, such as acute myocardial infarction, thromboembolic events, or initially reported COVID-19 outcomes, compared with non-smokers. The most widely accepted explanation for the smoking paradox is that it reflects reporting biases, cohort-specific factors, or unmeasured confounders rather than a true protective effect [41,42]. Conversely, participants reporting only occasional physical activity were more often symptomatic and hospitalized, suggesting that sporadic exercise may not provide the protective effects associated with regular physical activity [43,44]. These results support prior evidence linking consistent physical activity to milder COVID-19 outcomes and reduced hospitalization [45].

Cardiometabolic comorbidities (including diabetes, hypertension, hypercholesterolemia, obesity, and cardiovascular disease) were more prevalent among COVID-19 cases, particularly among hospitalized symptomatic patients. Although not all differences were statistically significant, these patterns are consistent with population-based studies and meta-analyses reporting an association between cardiometabolic conditions and symptomatic and severe COVID-19, hospitalization, and mortality [46,47,48,49].

Beyond these established determinants related to COVID-19, we further investigated the association of Neanderthal-derived genetic variants within the *OAS3* and *LZTFL1* genes with COVID-19 severity, with a particular focus on genotype-specific and combined effects. While allele-based analyses did not reveal statistically significant differences, some genotype-dependent patterns emerged, underscoring the potential limitations of single-variant approaches in capturing complex genetic effects.

This study reports, for the first time, allele frequencies of the examined genes in the Republic of Srpska, Bosnia and Herzegovina, and, to our knowledge, in the Balkan region overall, representing a unique contribution to the characterization of the genetic architecture of Balkan populations. The lack of comparable studies from other Balkan countries limits the regional contextualization of these results. Nevertheless, comparison of allele frequencies with those reported in reference databases for European populations revealed no significant deviations, indicating allele stability across human populations [50]. This observation suggests that the examined alleles are functionally relevant to human phenotypes and have likely been maintained through evolutionary time.

Zeberg and Pääbo have highlighted a region within the *OAS* gene cluster, encompassing all three *OAS* genes, as a protective Neanderthal haplotype potentially relevant to COVID-19 [15]. Their analysis of disease-associated SNPs indicated that while the broader genetic signal spans all OAS genes, the strongest associations were localized to *OAS3*, suggesting a particularly important role [15]. Subsequent genome-wide association studies (GWASs) and transcriptome-wide association studies (TWASs) have further supported these findings by implicating *OAS3* in antiviral activity and a protective response to SARS-CoV-2 infection [51,52]. Functionally, the OAS family comprises OAS1, OAS2, and OAS3, nucleotidyltransferases characterized by their ability to detect exogenous nucleic acids [20,22]. Upon viral entry into a host cell, viral RNA, which is distinct from host RNA, is recognized by specific receptors, triggering activation of OAS enzymes [53]. Activated OAS proteins convert ATP into 2′-5′-oligoadenylates (2–5 A), small signaling molecules that in turn activate latent ribonuclease L (RNase-L) [53]. RNase-L degrades both viral and cellular RNA, establishing an antiviral state that inhibits viral replication and can induce the death of infected cells, thereby limiting viral spread [53]. Although all OAS family members mediate antiviral defense, experimental models have shown that OAS3 exhibits a distinct activity spectrum compared with OAS1 and OAS2, whereas OAS1 can activate RNase L at lower concentrations of 2–5A, OAS3 typically acts as the primary viral RNA sensor and the main driver of the antiviral response [21,22]. Beyond their primary immunoregulatory role, OAS proteins have also been implicated in other cellular processes, sush as apoptosis, a key mechanism in tumor suppression and one of the ways by which the host responds to viral infection in an effort to eliminate virus-infected cells [54]. The high degree of linkage disequilibrium across the *OAS* locus may help explain why some studies have failed to replicate associations initially reported by Zeberg and Pääbo [16,17]. Because multiple variants are frequently inherited together, observed statistical associations may reflect the combined effects of several linked polymorphisms rather than the impact of a single functional change [15]. This may explain why SNP such as rs1156361 may show a significant genotype-specific association in certain cohorts but not in others, as the effect likely depends on a particular combination of linked variants present within each population.

Within this broader context, the absence of an association between *OAS3* rs1156361 and COVID-19 severity in our cohort is consistent with findings from other populations, including studies conducted in the Sardinia population [16,17]. Together with evidence from analyses of other *OAS3* variants—such as the lack of association of rs10735079 with disease severity in Morocco and the modest antiviral effects observed in functional overexpression studies—these results suggest that the contribution of *OAS3* to COVID-19 outcomes is subtle and likely dependent on broader genetic or regulatory context rather than individual variants alone [55,56]. Importantly, the presence of *OAS3* loss-of-function variants was not associated with more severe respiratory outcomes.

Neanderthal-derived variants within the chromosome 3p21.31 locus, particularly those encompassing the *LZTFL1* gene, have consistently emerged as some of the strongest genetic determinants of COVID-19 severity in large genome-wide association studies [18,19,57,58]. The risk haplotype at this locus has been associated with an approximately twofold increased risk of respiratory failure in COVID-19 patients [52,58]. However, identifying the specific causal variants underlying GWAS signals presents significant challenges. This limitation complicates interpretation and hinders the establishment of a direct causal relationship between genotype and COVID-19 severity. The analyzed SNPs are located in upstream or downstream regulatory regions of the *LZTFL1* gene and within enhancer elements, suggesting potential effects on RNA splicing, transcript diversity, and gene expression regulation. The possible involvement of alternative promoters further adds to the complexity of elucidating the molecular mechanisms underlying phenotype development. Although the biological mechanisms through which *LZTFL1* influences COVID-19 severity are not yet fully understood, evidence from TWASs and phenome-wide association studies (PheWASs) has identified lung tissue and lung function as key mediators of the COVID-19 association with the 3p21.31 locus [19,52]. Consistent with these findings, LZTFL1 is highly expressed in lung tissue, particularly in the respiratory epithelium, with predominant expression in ciliated epithelial cells [59,60]. Functionally, *LZTFL1* encodes a ubiquitously expressed cytoplasmic protein that regulates protein trafficking to the ciliary membrane through interactions with Bardet–Biedl syndrome protein complexes, underscoring its importance in epithelial organization and signaling [13,14]. Respiratory epithelial cells serve as the first line of defense against pathogens such as SARS-CoV-2, providing a physical barrier while actively contributing to the initiation and coordination of immune responses in the lungs [13,14]. When this barrier is compromised, viral entry is facilitated, leading to enhanced inflammation and, in severe cases, the development of a cytokine storm that can result in severe pneumonia and acute respiratory distress [13,57]. Although increased expression of this gene in lung epithelial cells may initially appear protective, in the context of COVID-19, it can instead impair epithelial regeneration and hinder normal recovery following viral injury [13,14,57]. In addition to its direct effects on the epithelium, genetic variants within the *LZTFL1* locus have been associated with altered immune cell migration, particularly T-lymphocyte recruitment, to lung tissue [14,57]. It is suggested that the combination of epithelial dysfunction and excessive immune activation underlies the development of severe forms of COVID-19, which often require hospitalization [13,14,57]. This gene also functions as a tumor suppressor, likely through modulation of epithelial–mesenchymal transition (EMT) [13,14]. EMT enables differentiated epithelial cells to acquire mesenchymal characteristics and plays an important role in innate immune responses [57]. Notably, SARS-CoV-2 has been shown to induce EMT in lung cancer cell lines as well as in respiratory epithelial cells [61,62]. Elevated *LZTFL1* expression may delay the beneficial effects of EMT by preventing the downregulation of ACE2 and TMPRSS2 and/or slowing EMT-driven tissue repair [57]. While current evidence links higher *LZTFL1* levels to worse outcomes in SARS-CoV-2 infection, further studies are needed to clarify its role and the contribution of EMT in lung pathogenesis.

Analysis of *LZTFL1* rs35044562 in our study demonstrated a complementary pattern of genotype-specific effects. The AA genotype was significantly more frequent among non-hospitalized individuals, suggesting a protective effect, whereas the AG genotype was significantly overrepresented among hospitalized patients. The difference in A and G allele frequencies did not reach statistical significance, again indicating that genotype-specific effects may be more informative than allele-based analyses alone.

While these findings have been replicated in some European populations, their interpretation must be approached with caution, due to population-specific genetic backgrounds. For example, Mocci et al. studied these polymorphisms in an isolated Sardinian population, which is characterized by a relatively limited genetic influx due to historical isolation, which limits generalizability to broader European populations [16,17]. On the other hand, Hubaček et al. analyzed *LZTFL1* rs35044562 and compared the majority Czech population with the non-Indigenous, highly mobile and heterogeneous Central European Roma population, highlighting how local genetic structure can influence interpretation [63]. Although associations between *LZTFL1* polymorphisms and COVID-19 severity have been reported across ethnically diverse populations, many of these studies examined different *LZTFL1* variants than rs35044562 analyzed in this study, limiting direct comparability. Nevertheless, evidence from independent cohorts, including the study by Angulo-Aguado et al. in the Colombian population and the analysis by Rüter et al., is consistent with our findings and supports a contributory role of *LZTFL1* COVID-19 disease severity [64,65].

Notably, the most pronounced association in our study was observed when the two loci were analyzed together. The concurrent presence of the CT genotype at *OAS3* rs1156361 and the AG genotype at *LZTFL1* rs35044562 was more frequent among hospitalized patients, although these findings should be interpreted cautiously. A similar analysis was conducted by Mocci et al.; however, they did not observe any significant associations between the combined *OAS3* and *LZTFL1* variants and COVID-19 outcomes. Given the lack of correlation or linkage disequilibrium between these variants, the observation is unlikely to reflect shared ancestry or haplotypic structure. Instead, it supports the possibility of an epistatic or synergistic interaction between biological pathways involved in early antiviral defense and those regulating epithelial integrity. A combined effect of compromised interferon-mediated viral clearance and increased vulnerability of the respiratory epithelium offers a biologically plausible explanation for the observed association with severe COVID-19, but further studies are required to confirm this potential interaction.

## 5. Conclusions

Our findings highlight the complex genetic architecture underlying COVID-19 severity, which cannot be fully captured by single-locus or allele-based analyses. The observed associations between *LZTFL1* variants and COVID-19 severity underscore the importance of integrative genetic approaches and warrant validation in larger, independent cohorts before definitive conclusions can be drawn. While these analyses remain exploratory and primarily serve to generate hypotheses, they offer important insights into the biological mechanisms influencing COVID-19 outcomes. Importantly, given the impact of the COVID-19 pandemic, our study carries some public health implications. By identifying population-specific genetic risk profiles, it may inform targeted interventions such as genetic screening of high-risk groups and support strategies for personalized prevention. Integrating these insights into prevention programs, health education initiatives, and resource allocation can enhance pandemic preparedness, reduce disease burden, and improve overall population health. Further research in diverse cohorts is essential to validate these findings and guide evidence-based policy and clinical practice.

## 6. Limitations

Several limitations of this study should be acknowledged. The relatively small and uneven number of participants across groups may have reduced statistical power to detect modest associations, particularly at the allele level and under recessive inheritance models. Consequently, these results should be interpreted with caution and considered exploratory, requiring validation in larger, independent cohorts. Nevertheless, this sample reflects the actual epidemiological situation at the time of sampling. Conducted alongside a population-based seroprevalence survey showing nearly 90% SARS-CoV-2 seroprevalence in the Republic of Srpska, the limited proportion of seronegative individuals aligns with population-level exposure patterns and supports the representativeness of the study sample.

Additionally, disease severity was assessed primarily through hospitalization status, which may be influenced by healthcare access, clinical decision-making, and local treatment protocols. During the study period, pandemic-related restrictions and lockdown measures substantially limited access to hospitalized patients. As a result, some of the most severe COVID-19 cases, particularly those requiring intensive care, may not have been included in the study, potentially leading to an underrepresentation of the most critical disease phenotypes. However, hospitalization criteria were primarily based on standardized clinical indicators of disease progression, such as respiratory compromise and systemic involvement. Therefore, hospitalization served as a reliable indicator of moderate-to-severe COVID-19 in this population-based setting.

## Figures and Tables

**Table 1:** Sociodemographic, epidemiological and clinical characteristics among COVID-19 asymptomatic and symptomatic patients.

Variables	COVID-19 Asymptomatic (n = 77, 20.4%)		COVID-19 Symptomatic (n = 301, 79.6%)		Total (n = 378)		*p* *
	n	%	n	%	n	%	
Male gender	39	50.6	123	40.9	162	42.9	0.122 *
Age (M ± SD)	44.64 ± 18.78		48.32 ± 15.25		47.43 ± 16.10		0.073 **
8 to 34 years	24	31.2	53	17.6	77	20.4	0.019 *
35 to 49 years	19	24.7	108	35.9	127	33.6	
50 to 83 years	34	44.2	140	46.5	174	46.0	
BMI (kg/m^2^) (M ± SD)	26.09 ± 4.66		26.60 ± 3.25		26.54 ± 3.41		0.656 **
High level of education	10	13.0	100	33.2	110	29.1	<0.001 *
Regularly employed	25	32.5	167	55.5	192	50.8	<0.001 *
Life in urban environment	26	33.8	199	66.1	225	59.5	<0.001 *
SARS-CoV-2 IgG-positive	22	75.9	147	91.3	169	88.9	0.015 *
Smoking	16	20.8	71	23.6	87	23.0	0.601*
Occasional sports activities	12	15.6	83	27.6	95	25.1	0.019 *
Comorbidities (yes)							
Diabetes mellitus	8	10.4	33	11.0	41	10.8	0.885 *
Hypertension	19	24.7	100	33.2	119	31.5	0.150 *
Hypercholesterolemia	9	11.7	65	21.6	74	19.6	0.050 *
Obesity	12	15.6	89	29.6	101	26.7	0.013 *
CVD	9	11.7	42	14.0	51	13.5	0.604 *
Cerebrovasc. dis.	0	0.0	3	1.0	3	0.8	0.379 *
Malignancies	1	1.3	7	2.3	8	2.1	0.576 *
CKDs	0	0.0	3	1.0	3	0.8	0.379 *
CLD	0	0.0	2	0.7	2	0.5	0.473 *
COPD	1	1.3	11	3.7	12	3.2	0.293 *
Autoimmune diseases	1	1.3	10	3.3	11	2.9	0.346 *
Genetic diseases in the family	1	1.3	10	3.3	11	2.9	0.346 *

BMI—body mass index; SARS-CoV-2 -severe acute respiratory syndrome coronavirus 2; CVDs—cardiovascular diseases; Cerebrovasc. dis.s—cerebrovascular diseases; CKDs—chronic kidney diseases; CLD—chronic liver disease; COPD—chronic obstructive pulmonary disease; M—mean ± SD—standard deviation, *p*—statistical significance was measured by * χ^2^—chi square test or Fisher’s exact test and ** Mann–Whitney test, significant values are bolded.

**Table 2:** Sociodemographic, epidemiological and clinical characteristics among symptomatic COVID-19 nonhospitalized and hospitalized patients.

Variables	COVID-19 Nonhospitalized (n = 225, 74.8%)		COVID-19 Hospitalized (n = 76, 25.2%)		Total (n = 301)		*p* *
	n	%	n	%	n	%	
Male gender	83	36.9	40	52.6	123	40.9	0.016 *
Age (M ± SD)	45.27 ± 15.04		57.33 ± 12.03		48.32 ± 15.25		<0.001 **
8 to 34 years	50	22.2	3	3.9	53	17.6	<0.001 *
35 to 49 years	89	39.6	19	25.0	108	35.9	
50 to 83 years	86	38.2	54	71.1	140	46.5	
BMI (kg/m^2^) (M ± SD)	25.86 ± 3.24		28.03 ± 2.79		26.60 ± 3.25		0.005 **
High level of education	78	34.7	22	28.9	100	33.2	0.360 *
Regularly employed	136	60.4	31	40.8	167	55.5	0.003 *
Life in urban environment	147	65.3	52	68.4	199	66.1	0.623 *
SARS-CoV-2 IgG-positive	117	90.7	30	93.8	147	91.3	0.583 *
Smoking	63	28.0	8	10.5	71	23.6	0.002 *
Occasional sports activities	65	28.9	18	23.7	83	27.6	0.380 *
Comorbidities (yes)							
Diabetes mellitus	18	8.0	15	19.7	33	11.0	0.005 *
Hypertension	56	24.9	44	57.9	100	33.2	0.000 *
Hypercholesterolemia	40	17.8	25	32.9	65	21.6	0.006 *
Obesity	56	24.9	33	43.4	89	29.6	0.002 *
CVD	22	9.8	20	26.3	42	14.0	0.000 *
Cerebrovasc. dis.	2	0.9	1	1.3	3	1.0	0.746 *
Malignancies	6	2.7	1	1.3	7	2.3	0.499 *
CKD	2	0.9	1	1.3	3	1.0	0.746 *
CLD	1	0.4	1	1.3	2	0.7	0.419 *
COPD	6	2.7	5	6.6	11	3.7	0.116 *
Autoimmune diseases	10	4.4	0	0.0	10	3.3	0.062 *
Genetic diseases in the family	6	2.7	4	5.3	10	3.3	0.275 *

BMI—body mass index; SARS-CoV-2 -severe acute respiratory syndrome coronavirus 2; CVD—cardiovascular diseases; Cerebrovasc. Dis.—cerebrovascular diseases; CKD—chronic kidney diseases; CLD—chronic liver disease; COPD—Chronic obstructive pulmonary disease; M—mean ± SD—standard deviation, *p*—statistical significance was measured by * χ^2^—chi square test or Fisher’s exact test and ** Mann-Whitney test, significant values are bolded.

**Table 3:** Frequencies of *OAS3* rs1156361 and *LZTFL1* rs35044562 genotypes and alleles in COVID-19 asymptomatic and symptomatic patients and their association with the risk of occurrence of symptoms.

Genotypes and Alleles	COVID-19 Asymptomatic (n = 77, 20.4%)	COVID-19 Symptomatic (n = 301, 79.6%)	Total n (%)	*p* *	Adjusted Logistic Regression Analysis		
					AOR Value	95% CI	*p* **
**SNP OAS 3** **rs1156361**							
CC (%)	28 (36.4)	135 (44.9)	163 (43.1)	0.180	-	-	Referent
CT (%)	39 (50.6)	133 (44.2)	172 (45.5)	0.309	0.883	0.201–1.993	0.779
TT (%)	10 (13.0)	33 (11.0)	43 (11.4)	0.618	0.562	0.091–1.735	0.987
C	95 (61.7)	403 (66.9)	498 (65.9)				
T	59 (38.3)	199 (33.1)	258 (34.11)	0.223			
**SNP LZTFL1** **rs35044562**							
AA (%)	53 (68.8)	222 (73.8)	275 (72.8)	0.387	-	-	Referent
AG (%)	24 (31.2)	75 (24.9)	99 (26.2)	0.266	0.890	0.403–1.839	0.901
GG (%)	0 (0.0)	4 (1.3)	4 (1.1)	0.309	0.289	0.073–0.881	0.810
A	130 (84.4)	519 (86.2)	649 (85.8)				
G	24 (15.6)	83 (13.8)	107 (14.2)	0.566			

AOR—adjusted odds ratio; 95% CI—confidence interval; *p* *—statistical significance for Chi-square test; *p* **—statistical significance for multivariate analysis adjusted for univariate significant variables; significant values are bolded.

**Table 4:** Frequencies of *LZTFL1* rs35044562 and *OAS* rs1156361 genotypes and alleles in COVID-19 nonhospitalized and hospitalized COVID-19 positive symptomatic patients and their association with the hospitalization risk.

Genotypes and Alleles	COVID-19 Nonhospitalized (n = 225, 74.8%)	COVID-19 Hospitalized (n = 76, 25.2%)	Total n (%)	*p* *	Adjusted Logistic Regression Analysis		
					AOR Value	95% CI	*p* **
**SNP OAS3** **rs1156361**							
CC (%)	103 (45.8)	32 (42.1)	135 (44.8)	0.578	-	-	Referent
CT (%)	95 (42.2)	38 (50.0)	133 (44.2)	0.238	0.891	0.293–3.201	0.693
TT (%)	27 (12.0)	6 (7.9)	33 (11.0)	0.322	0.204	0.078–0.991	0.909
C	301 (66.9)	102 (67.1)	403 (66.9)				
T	149 (33.1)	50 (32.9)	199 (33.1)	0.974			
**SNP *LZTFL1*** **rs35044562**							
AA (%)	174 (77.3)	48 (63.2)	222 (73.8)	**0.015**	-	-	Referent
AG (%)	47 (20.9)	28 (36.8)	75 (24.9)	**0.005**	1.372	0.763–6.383	**0.021**
GG (%)	4 (1.8)	0 (0.0)	4 (1.3)	0.242	0.293	0.092–0.993	0.982
A	395 (87.8)	124 (81.6)	519 (86.2)				
G	55 (12.2)	28 (18.4)	83 (13.8)	0.058			

AOR—adjusted odds ratio; 95% CI—confidence interval; *p* *—statistical significance for Chi-square test; *p* **—statistical significance for multivariate analysis adjusted for univariate significant variables; significant values are bolded.

**Table 5:** Association of COVID-19 hospitalization risk with *LZTFL1* rs35044562 polymorphisms in an allele model.

*LZTFL1* rs35044562 Genotypes	COVID-19 Nonhospitalized (n = 225, 74.8%)	COVID-19 Hospitalized (n = 76, 25.2%)	Total n (%)	*p* *	Adjusted Logistic Regression Analysis		
					OR Value	95% CI	*p* **
							
AA (%)	174 (77.3)	48 (63.2)	222 (73.8)				
AG + GG (%)	51 (22.7)	28 (36.8)	79 (26.2)	**0.015**	1.101	0.690–4.229	**0.022**
							
AG (%)	47 (20.9)	28 (36.8)	75 (24.9)				
AA + GG (%)	178 (79.1)	48 (63.2)	226 (75.1)	**0.001**	0.892	0.301–2.240	0.142
							
GG (%)	4 (1.8)	0 (0.0)	4 (1.3)				
AA + AG (%)	221 (98.2)	76 (100.0)	297 (98.7)	0.575	0.339	0.104–1.289	0.303

AOR—adjusted odds ratio; 95% CI—confidence interval; *p* *—statistical significance for Chi-square test; *p* **—statistical significance for multivariate analysis adjusted for univariately significant variables; significant values are bolded.

**Table 6:** Frequencies of *OAS3* rs1156361 and *LZTFL1* rs35044562 genotypes and alleles in healthy individuals and COVID-19 patients and their association with susceptibility to SARS-CoV-2 infection.

Genotypes and Alleles	Healthy Individuals (n = 24, 5.9%)	COVID-19 Positive (n = 378, 94.1%)	Total n (%)	*p* *	Adjusted logistic Regression Analysis		
					AOR Value	95% CI	*p* *
**SNP *OAS 3*** **rs1156361**							
CC (%)	15 (62.5)	163 (43.1)	178 (44.3)	0.064	-	-	Referent
CT (%)	6 (25.0)	172 (45.5)	178 (44.3)	**0.050**	0.802	0.239–3.663	**0.043**
TT (%)	3 (12.5)	43 (11.4)	46 (11.4)	0.867	0.227	0.049–1.227	0.982
C	36 (75.0)	498 (65.9)	534 (66.4)				
T	12 (25.0)	258 (34.1)	270 (33.6)	0.193			
**SNP *LZTFL1*** **rs35044562**							
AA (%)	17 (70.8)	275 (72.8)	292 (72.6)	0.838	-	-	Referent
AG (%)	7 (29.2)	99 (26.2)	106 (26.4)	0.748	0.669	0.203–1.9775	0.902
GG (%)	0 (0.0)	4 (1.1)	4 (1.0)	0.613	0.776	0.391–1.203	0.978
A	41 (85.4)	649 (85.9)	690 (85.8)				
G	7 (14.6)	107 (14.1)	114 (14.2)	0.941			

AOR—adjusted odds ratio; 95% CI—confidence interval; p*—statistical significance for Chi-square test; p**—statistical significance for multivariate analysis adjusted for univariate significant variables; significant values are bolded.

**Table 7:** Association between *OAS3* rs1156361 polymorphisms and susceptibility to SARS-CoV-2 infection in an allelic model analysis.

OAS3 rs1156361 Genotypes	Healthy Individuals (n = 24, 5.9%)	COVID-19 Positive (n = 378, 94.1%)	Total n (%)	*p* *	Adjusted Logistic Regression Analysis		
					AOR Value	95% CI	*p* **
							
CC (%)	15 (62.5)	163 (43.1)	178 (44.3)				
CT + TT (%)	9 (37.5)	215 (56.9)	224 (55.7)	0.064	0.301	0.102–0.996	0.779
							
CT (%)	6 (25.0)	172 (45.5)	178 (44.3)				
CC + TT (%)	18 (75.0)	206 (54.5)	224 (55.7)	**0.049**	0.799	0.199–3.702	**0.033**
							
TT (%)	3 (12.5)	43 (11.4)	46 (11.4)				
CC + CT (%)	21 (87.5)	335 (88.6)	356 (88.6)	0.992	0.692	0.403–1.227	0.992

AOR—adjusted odds ratio; 95% CI—confidence interval; *p* *—statistical significance for Chi-square test; *p* **—statistical significance for multivariate analysis adjusted for univariate significant variables; significant values are bolded.

## Data Availability

The original contributions presented in this study are included in the article/Supplementary Materials. Further inquiries can be directed to the corresponding author.

## Supplementary Materials

The following supporting information can be downloaded at: https://www.mdpi.com/article/10.3390/biomedicines14020478/s1. Table S1: Sociodemographic, epidemiological and clinical characteristics among healthy individuals and COVID-19 positive patients; Table S2: Hardy–Weinberg equilibrium (HWE) in the COVID-19 positive patients; Table S3: Comparison of obtained allele frequencies with the allele frequencies for the European population; Table S4: Association between OAS3 rs1156361 and LZTFL1 rs35044562 polymorphism combinations and COVID-19 hospitalization risk.
