# Supplementary material for: Genetic, Sociodemographic and Clinical Determinants of COVID-19 Severity in the Republic of Srpska: Exploring Potential Links with Neanderthal-Derived Variants
Source: Biomedicines. 2026 Feb 22;14(2):478. doi: 10.3390/biomedicines14020478 (PMC12937615; doi:10.3390/biomedicines14020478)
Supplement: Supplementary file 1 [file biomedicines-14-00478-s001.zip › biomedicines-4135343-supplementary.pdf]

Supplementary Table S1. Socio-demographic, epidemiological and clinical characteristics among healthy individuals and COVID-19 positive patients.

Variables	Healthy individuals (n=24, 5.9%)		COVID-19 positive (n=378, 94.1%)		Total (n=402)		*p**
	n	%	n	%	n	%	
Male gender	16	66.7	162	42.8	178	44.3	**0.023***
Age (M±SD)	48.04±18.27		47.43±16.10		47.6±16.21		0.745**
8 to 34 years	7	29.2	77	20.4	84	20.9	
35 to 49 years	4	16.7	127	33.6	131	32.6	0.208*
50 to 83 years	13	54.1	174	46.0	187	46.5	
BMI (kg/m^2) (M±SD)	27.18±3.26		26.54±3.41		26.57±3.39		0.715**
High level of education	3	12.5	110	29.1	113	28.1	0.079*
Regularly employed	10	41.7	192	50.8	202	50.2	0.386*
Life in urban environment	12	50.0	225	59.5	237	59.0	0.358*
SARS-CoV-2 IgG positive	0	0.0	169	88.9	169	84.9	**<0.001***
Smoking	7	29.2	87	23.0	94	23.4	0.490*
Occasional sports activities	5	20.8	95	25.1	100	24.9	0.637*
Commorbidities (yes)							
Diabetes mellitus	0	0.0	41	10.8	41	10.2	0.089*
Hypertension	4	16.7	119	31.5	123	30.6	0.127*
Hypercholesterolemia	2	8.3	74	19.6	76	18.9	0.173*
Obesity	6	25.0	101	26.7	107	26.6	0.848*
CVD	2	8.3	51	13.5	53	13.2	0.469*
Cerebrovasc. Dis.	0	0.0	3	0.8	3	0.7	0.661*
Malignancies	0	0.0	8	2.1	8	2.0	0.472*
CKD	0	0.0	3	0.8	3	0.7	0.661*
CLD	0	0.0	2	0.5	2	0.5	0.721*
COPD	0	0.0	12	3.2	12	3.0	0.376*
Autoimmune diseases	0	0.0	11	2.9	11	2.7	0.397*
Genetic diseases in the family	1	4.2	11	2.9	12	3.0	0.726*

BMI – body mass index; SARS-CoV-2 -severe acute respiratory syndrome coronavirus 2; CVD – cardiovascular diseases; Cerebrovasc. Dis. – cerebrovascular diseases; CKD – chronic kidney diseases; CLD – chronic liver disease; COPD - Chronic obstructive pulmonary disease; M - mean ± SD - standard deviation, p – statistical significance was measured by *χ^2 – chi square test or Fisher's exact test and **Mann-Whitney test, significant values are bolded

Supplementary Table S2. Hardy–Weinberg equilibrium (HWE) in the COVID-19 positive patients.

Gene	SNP		Expected value	Observed value
			COVID-19 positive	COVID-19 positive
OAS3	rs1156361(T>C)	Genotype		
		CC	164.02	163

	CT	169.95	172
	TT	44.02	43
	Allele frequency		
	C	0.6587	
	T	0.34413	
	χ2 value	0.0549	
	p-value	0.8148	
LZTFL1 rs35044562(A>G)	Genotype		
	AA	278.57	275
	AG	91.86	99
	GG	7.57	4
	Allele frequency		
	A	0.8585	
	G	0.1415	
	χ2 value	2.2866	
	p-value	0.1305	

p – statistical significance was measured by *χ^2 – chi square test or Fisher's exact test; significant values are bolded.*

Supplementary Table S3. Comparation of obtained allele frequencies with the allele frequencies for the European population from referent database*.

Gene SNP		Observed value	Expected value	
		Analysed population	European population	χ2 value *p-value*
OAS3 rs1156361(T>C)	Allele frequency			
	C	0,664	0,64035	0,0009 *0,976*
	T	0,336	0,35965	0,0016 *0,969*
LZTFL1 rs35044562(A>G)	Allele frequency			χ2 value *p-value*
	A	0,858	0,91889	0,0040 *0,949*
	G	0,142	0,08111	0,0457 *0,831*

*https://www.ncbi.nlm.nih.gov/snp/rs1156361 and https://www.ncbi.nlm.nih.gov/snp/rs35044562

p – statistical significance was measured by *χ^2 – chi square test or Fisher's exact test.*

Supplementary Table S4. Association between *OAS3* rs1156361 and *LZTFL1* rs35044562 polymorphism combinations and COVID-19 hospitalization risk.

OAS3 rs1156361 genotypes	*LZTFL1* rs35044562 genotypes	**COVID-19 nonhospitalized** (n=225, 74.8%)	**COVID-19 hospitalized** (n=76, 25.2%)	**Total** (n=301)	*p**
		n %	n %	n %	
**CC**	**AA**	73 (32.4)	20 (26.3)	93 (30.9)	0.317
**CT**	**AA**	79 (35.1)	24 (31.6)	103 (34.2)	0.575
**TT**	**AA**	23 (10.2)	4 (5.3)	27 (9.0)	0.191
**CC**	**AG**	26 (11.6)	12 (15.8)	38(12.6)	0.337
**CT**	**AG**	14 (6.2)	14 (18.4)	28 (9.3)	**0.020**
**TT**	**AG**	5 (2.2)	2 (2.6)	7 (2.3)	0.838
**CC**	**GG**	3 (1.3)	0 (0.0)	3 (1.0)	0.312
**CT**	**GG**	1 (0.4)	0 (0.0)	1 (0.3)	0.560
**TT**	**GG**	0 (0.0)	0 (0.0)	0 (0.0)	

p – statistical significance was measured by *χ^2 – chi square test or Fisher's exact test; significant values are bolded.*
